# Patient-derived iPSC models of Friedreich ataxia: a new frontier for understanding disease mechanisms and therapeutic application

**DOI:** 10.1186/s40035-023-00376-8

**Published:** 2023-09-20

**Authors:** Saumya Maheshwari, Gabriela Vilema-Enríquez, Richard Wade-Martins

**Affiliations:** Department of Physiology, Anatomy and Genetics, Kavli Institute for Nanoscience Discovery, University of Oxford, South Parks Road, Oxford, OX1 3QU UK

**Keywords:** Friedreich ataxia, Induced pluripotent stem cells, Disease modelling, Disease pathogenesis, Drug development

## Abstract

Friedreich ataxia (FRDA) is a rare genetic multisystem disorder caused by a pathological GAA trinucleotide repeat expansion in the *FXN* gene. The numerous drawbacks of historical cellular and rodent models of FRDA have caused difficulty in performing effective mechanistic and translational studies to investigate the disease. The recent discovery and subsequent development of induced pluripotent stem cell (iPSC) technology provides an exciting platform to enable enhanced disease modelling for studies of rare genetic diseases. Utilising iPSCs, researchers have created phenotypically relevant and previously inaccessible cellular models of FRDA. These models enable studies of the molecular mechanisms underlying GAA-induced pathology, as well as providing an exciting tool for the screening and testing of novel disease-modifying therapies. This review explores how the use of iPSCs to study FRDA has developed over the past decade, as well as discussing the enormous therapeutic potentials of iPSC-derived models, their current limitations and their future direction within the field of FRDA research.

**Graphical abstract:**

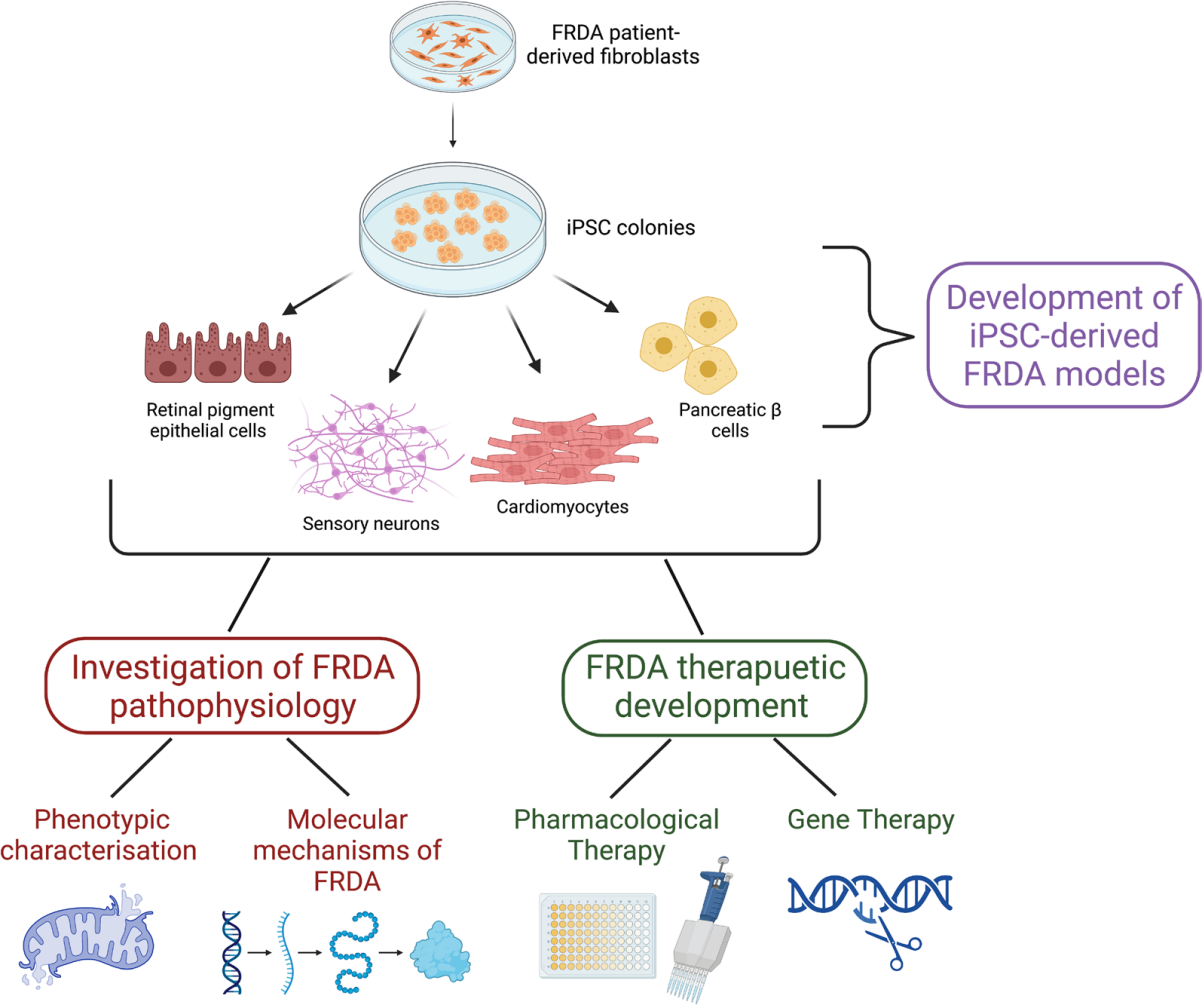

## Background

Friedreich ataxia (FRDA) is a rare genetic disorder of late childhood and the most common form of inherited ataxia, with an incidence of approximately 1 in 50,000 [1]. A GAA trinucleotide repeat expansion in the intronic region of the *FXN* gene is the pathological basis of the disorder, with the disease inherited in an autosomal recessive manner. The *FXN* gene is present on chromosome 9 and encodes frataxin, an essential mitochondrial protein expressed by all cells within the body. In FRDA, the GAA repeat expansion results in decreased production of frataxin, leading to impaired iron homeostasis, mitochondrial iron accumulation and eventually cell death [2]. Several cell types are particularly sensitive to reduced frataxin, particularly neurons within the central and peripheral nervous systems (CNS and PNS), hence the majority of FRDA sufferers experience progressive neurodegeneration during their lifetime. Signs classically begin between the ages of 5 and 15 and include progressive ataxia, dysarthria, spasticity, muscle weakness and loss of limb sensation. Between one to two decades following symptom onset, individuals commonly become wheelchair-bound. FRDA is a multisystem disorder, with many patients also developing hypertrophic cardiomyopathy, aggressive scoliosis and overt diabetes, impacting their quality of life and reducing life-expectancy [3]. Despite the progressive and inherently disabling nature of FRDA, the disorder remains without a cure, causing substantial interest within the scientific community to develop a therapy which may halt and potentially reverse the FRDA phenotype. This has proven a challenging proposition, likely due to an incomplete understanding of the underlying disease pathogenesis, alongside inadequate access to effective platforms which enable drug development and discovery.

The inability to access the most phenotypically relevant cell types affected by FRDA, being sensory neurons, cardiomyocytes and pancreatic β cells, is a major barrier to unravelling the complex molecular mechanisms underpinning disease progression. This has led to a historic dependency on several animal models to perform mechanistic and therapeutic studies [4]. While animal models have been useful to progress our understanding of the disease, their inability to adequately recapitulate disease phenotype has led researchers to seek a more sophisticated cellular model to investigate FRDA.

Substantial progress in the field of stem cell technology and the recent advent of iPSCs have provided researchers with a promising cellular tool to probe FRDA. First described by Yamanaka in 2006 [5], iPSCs are similar in many ways to embryonic stem cells (ESCs), possessing the potential to propagate indefinitely in vitro and differentiate into mature cell types derived from any of the embryonic germ layers. However, iPSCs are obtained by cellular reprogramming of somatic cells via the overexpression of key transcription factors (typically, OCT4, SOX2, C-MYC and KLF4) to induce pluripotency. The ability to generate iPSCs directly from mature somatic cells such as patient fibroblast lines bypasses many key ethical issues which surround the generation and supply of ESCs [6].

As technology within this field has rapidly progressed, laboratories worldwide have optimised the reprograming process and developed iPSC differentiation protocols to generate many neuronal phenotypes most commonly affected by neurogenerative diseases. These include dopaminergic, GABAergic, glutamatergic and motor neurons [7]. The ability to utilise patient-specific somatic cells for iPSC generation followed by the subsequent differentiation of disease-specific neuronal cell lines is of particular interest. These neurons possess the same genetic background as affected patient cells, providing a crucial platform to decipher disease mechanisms and screen novel therapeutic avenues. As reprogramming techniques and differentiation protocols have been developed, there has been increasing interest to utilise iPSC technology to investigate rarer genetic disorders. FRDA, a disorder affecting many inaccessible cell types, represents a model disease for which the use of iPSCs may provide exciting breakthroughs in mechanistic studies and drug discovery programs (see Graphical Abstract).

## Establishing iPSC-derived models for the investigation of FRDA pathology

### Early FRDA iPSC studies and creation of relevant cellular models from FRDA iPSCs

**Fig. 1:**
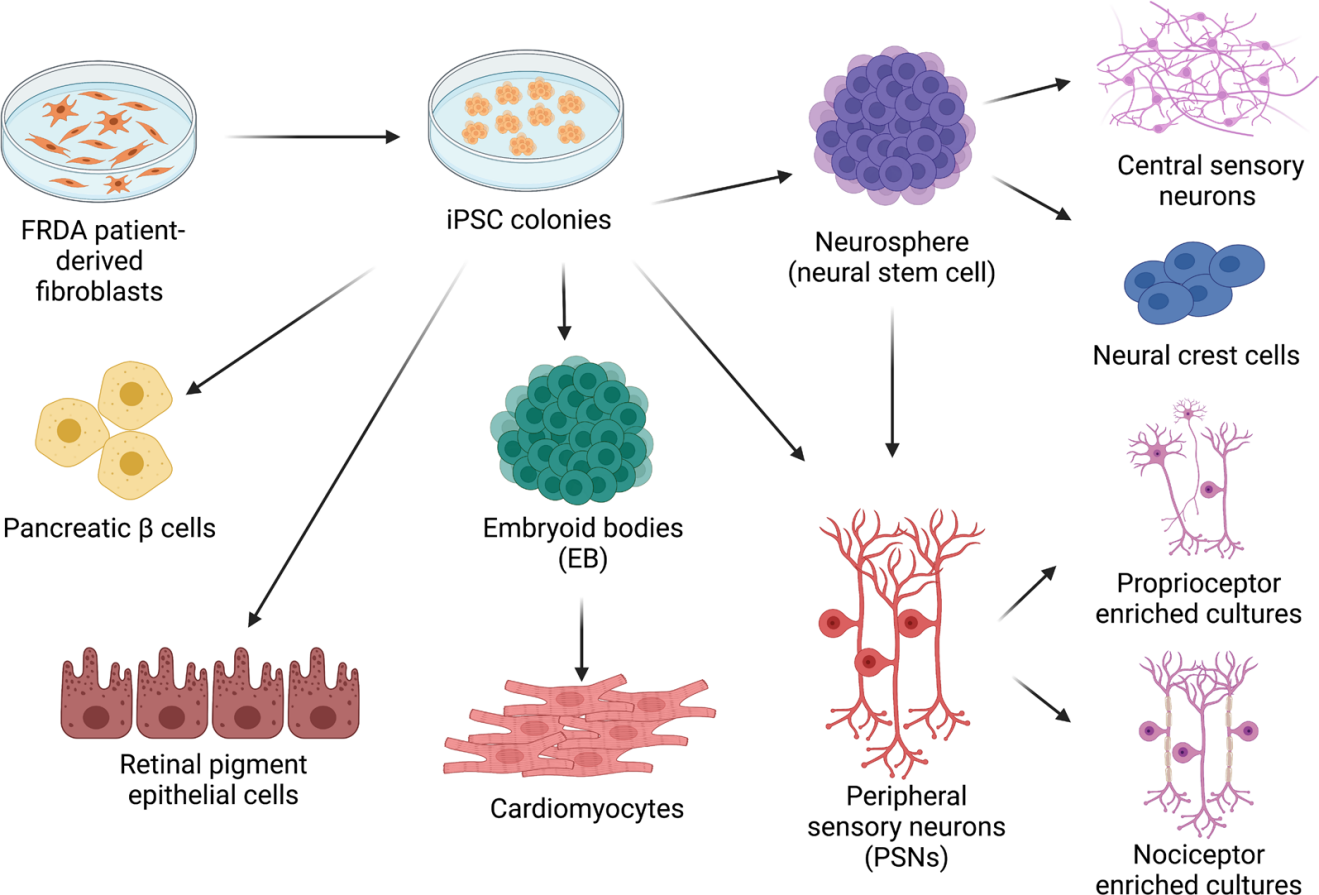
Cellular models derived from FRDA iPSCs. FRDA patient-derived fibroblasts are reprogrammed into iPSCs by cellular reprogramming through the overexpression of OCT4, SOX2, C-MYC and KLF4, transcription factors known to induce pluripotency. iPSC colonies are expanded, selected and confirmed to express pluripotency markers and to be karyotypically normal. At the iPSC stage, cells can be converted into almost any cell type. Particularly relevant for FRDA is the conversion to neurons, cardiomyocytes, pancreatic β cells and retinal pigment epithelial cells. Figure created with BioRender.com

Over the past decade, several differentiation protocols have been developed to generate more phenotypically relevant cell models from FRDA iPSCs. These include cell types significantly affected by the disease, such as neurons, cardiomyocytes and pancreatic β cells (Fig. 1).

The first study to generate FRDA iPSCs was performed in 2010, reprogramming 2 FRDA patient primary fibroblast lines to produce FRDA iPSCs [8]. The derived iPSCs lines not only maintained *FXN* gene repression but also exhibited similar patterns of repeat expansion and contraction across generations, as commonly described in human disease. The results of the study also crucially provided evidence for the role of mismatch repair (MMR) enzyme MSH2 in repeat instability, demonstrating the potential for FRDA iPSCs to elucidate the molecular mechanisms underpinning FRDA pathology.

One year later in 2011, neurons and cardiomyocytes were successfully generated from FRDA iPSCs [9], followed by a study published in 2013 demonstrating that mature cell types derived from FRDA iPSCs displayed a relevant frataxin deficiency phenotype [10]. Since the publication of these pioneering studies, several groups worldwide have developed differentiation protocols to generate various cellular models derived from FRDA iPSCs. These models have been used to investigate the molecular mechanisms driving GAA expansion instability in FRDA and allow for the exploration of novel therapeutic avenues. The following section will detail the development of these iPSC-derived models by cell type, as well as describing their use to study mechanisms underpinning FRDA pathology (see Table 1 for a summary of the studies described).

**Table 1:** Studies detailing the generation of FRDA iPSCs and their derived models, as well as their use to investigate FRDA pathology

Study theme	Study	Year	Cell type(s) utilised	Study outcome
Developing iPSC-derived cellular models of FRDA	Liu et al. [9]	2011	FRDA iPSC-derived peripheral neurons FRDA iPSC-derived cardiomyocytes	Successful generation of FRDA iPSCs from patient fibroblasts. These FRDA iPSCs could be differentiated into peripheral neurons and cardiomyocytes
	Wong et al*.* [11]	2019	FRDA iPSC-derived 3D human ventricular cardiomyocyte model	Generation of 3D human-engineered cardiac tissue models from FRDA iPSC-derived cardiomyocytes. These cardiac models show electrophysiological defects and *FXN* expression-dependent contractility defects
	Mazzara et al*.* [12]	2020	FRDA iPSC-derived dorsal root ganglia organoid sensory neurons	Generation of a DRG organoid-derived sensory neuronal model from FRDA iPSCs. This model exhibits molecular and cellular phenotypes which are reversed upon excision of the *FXN* intron 1
	Dionisi et al*.* [13]	2020	FRDA iPSC-derived primary proprioceptive neurons	Development of a protocol allowing the successful generation of proprioceptive enhanced cultures (up to 50% of finally differentiated neurons) from FRDA iPSCs. Further cell sorting with FACS resulted in almost pure proprioceptive cultures
Investigation of FRDA phenotypic characteristics	Hick et al*.* [10]	2013	FRDA iPSC-derived neurons FRDA iPSC-derived cardiomyocytes	FRDA iPSCs demonstrate expansion instability and reduced *FXN* expression, but no biological phenotypes. Subsequently derived neurons and cardiomyocytes demonstrate diseased mitochondrial phenotypes
	Lee et al. [14]	2014	FRDA iPSC-derived cardiomyocytes	FRDA iPSC-derived cardiomyocytes are similar in size, ATP production rate and calcium handling phenotypes when compared to wild-type controls, despite exhibiting some mitochondrial defects. The presence of an excessive iron supplement resulted in the display of iron-overloading cardiomyopathy phenotypes in the same cells
	Bird et al*.* [15]	2014	FRDA iPSC-derived neurons	FRDA iPSC-derived neurons possess normal mitochondrial function and show no altered susceptibility to cell death. FRDA iPSC-derived neural progenitors differentiate into functional neurons and following transplantation can successfully integrate in vivo in the cerebellum of adult rodents
	Crombie et al*.* [16]	2015	FRDA iPSC-derived retinal pigment epithelium cells	Retinal pigment epithelium cells derived from FRDA iPSCs display normal oxidative phosphorylation activity and normal phagocytosis
	Crombie et al. [17]	2017	FRDA iPSC-derived cardiomyocytes	FRDA iPSC-derived cardiomyocytes demonstrate electrophysiological phenotypes of calcium handling deficiency such as increased variation in beating rates (prevented with nifedipine) and low calcium transients
	Bolotta et al*.* [18]	2019	FRDA iPSC-derived cardiomyocytes	FRDA iPSC-derived cardiomyocytes exhibit increased protein expression of hepcidin and ferroportin and decreased levels of nuclear ferroportin in comparison to controls
Investigation into molecular mechanisms underpinning FRDA pathology	Ku et al*.* [8]	2010	FRDA iPSCs	Successful generation of iPSCs from FRDA patient fibroblasts, which maintain *FXN* gene repression and demonstrate GAA repeat instability. Silencing of *MSH2* (which occupies *FXN* intron 1) impairs the GAA repeat expansion in FRDA iPSCs
	Du et al. [19]	2012	FRDA iPSCs FRDA iPSC-derived neural precursors FRDA iPSC-derived neurospheres	Increased expression of *MSH2*, *MSH3* and *MSH6* was found in FRDA patient-derived iPSCs, with silencing of *MSH2* and *MSH6* impairing the repeat expansion. Treatment of FRDA iPSCs with polyamide FA1 partially blocks GAA repeat expansions
	Eigentler et al*.* [20]	2013	FRDA iPSC-derived peripheral sensory neurons	Successful generation of peripheral sensory neurons and neural crest progenitors from FRDA iPSCs. FRDA iPSCs failed to upregulate frataxin during differentiation to FRDA peripheral sensory neurons
	Shan et al*.* [21]	2014	FRDA iPSC-derived neural stem cells	Identification of protein targets and mechanistic pathways for an HDAC inhibitor (compound 106) in FRDA iPSC-derived neural stem cells. Targets of compound 106 are likely involved in both transcriptional regulation and post-transcriptional processing of mRNA
	Igoillo-Esteve et al*.* [22]	2015	FRDA iPSC-derived β cells FRDA iPSC-derived neurons	β cell death due to frataxin deficiency is a consequence of activation of the intrinsic apoptotic pathway, which is activated in FRDA iPSC-derived neurons and β cells. Prevention of the intrinsic apoptotic pathway activation is seen with cAMP induction
	Rodden et al*.* [23]	2021	FRDA iPSC-derived neurons	Determination of previously unrecognised differentially methylated region upstream of expanded repeat in FRDA iPSC-derived neurons
	Cotticelli et al*.* [24]	2022	FRDA iPSC-derived cardiomyocytes	Transcriptomic analysis of a novel FRDA iPSC isogenic cardiomyocyte model demonstrated mitochondrial dysfunction and a type 1 interferon activation response as pathways most affected by frataxin deficiency
	Angulo et al*.* [25]	2022	FRDA iPSC-derived neurons FRDA iPSC-derived cardiomyocytes	Identification of differentially expressed genes in FRDA iPSC-derived neurons and cardiomyocytes demonstrated that glycolysis and extracellular matrix-involved pathways are most affected by *FXN* deficiency in neurons and cardiomyocytes, respectively

FACS, fluorescent-activated cell sorting

### iPSC-derived neuronal cell models

FRDA predominately results in the localised neurodegeneration of cell types found in both the CNS and the PNS, including the dorsal root ganglia, posterior columns, cortical spinal tracts, peripheral sensory neurons and cerebellar neurons of the dentate nucleus and its connections [26]. Several of these neuronal cell types have been successfully generated from FRDA iPSCs (Fig. 1). Typically, studies have focused on the derivation of “default” central sensory neurons and neural crest cells. This process involves neural induction of iPSC colonies by noggin to form neural stem cells, which are propagated and maintained as neurospheres. These neural precursors can then be induced to differentiate into mature cell types via the addition/abstention of various factors within the culture medium. The development of patient-derived sensory neurons has provided researchers with a useful tool to investigate the phenotypic characteristics and biological processes driving FRDA disease pathology in a relevant neuronal model. One of the earliest studies to utilise this tool characterised pathological mitochondrial phenotypes in FRDA iPSC-derived sensory neurons. When compared to control iPSCs, neurons from FRDA iPSCs demonstrated a reduced mitochondrial membrane potential and a delayed development of electrophysiological functionality following differentiation, confirming a role of mitochondrial pathogenesis in FRDA and providing several cellular phenotypes for further study [10]. Interestingly, in 2014, a contrasting study was published showing no overt differences in the long-term survival and mitochondrial function in FRDA-derived neural progenitors and neurons, respectively, compared to control lines. The FRDA iPSCs were able to differentiate into fully functional neurons, with normal electrophysiological recording comparative to control lines. Additionally, no alteration in mitochondrial volume or function was seen in FRDA neurospheres, which also showed no increase in cell death compared to control iPSC- and human embryonic stem cell-derived neurospheres. This study was also the first to provide an in vivo characterisation of FRDA-derived neurons, showing that FRDA iPSC-derived neurospheres were able to integrate, differentiate and survive in rats [15]. This contrasting inability to detect overt phenotypic characteristics of neurodegeneration may be due to several factors relating to the underlying differences in the iPSC lines utilised, the immature nature of derived neurons and the late-childhood onset of degeneration seen in symptomatic disease. We shall discuss these factors in a later section of this review.

The differences in phenotypic presentation seen in cellular models may also be due to the heterogeneity of cells derived from FRDA iPSCs. In their studies, both Hick et al. [10] and Bird et al. [15] employed protocols to derive central sensory neurons, similar to immature cortical neurons. Neurodegeneration in FRDA is predominately seen to affect the peripheral sensory neurons (PSNs) which compose the dorsal root ganglion (DRG) of the spinal cord. These PSNs are more susceptible to decreased frataxin levels compared to central neurons, thus being disproportionality affected by FRDA pathology. With this in mind, several groups worldwide have worked to develop differentiation protocols for the derivation of more FRDA-specific neuronal cell models. The first of these models established was a development-based differentiation protocol to specifically generate neural-crest PSNs from FRDA-derived iPSCs. Utilising their novel model, the group was able to demonstrate an early failure to upregulate frataxin expression during sensory differentiation of FRDA PSNs, highlighting a potential developmental element to FRDA pathology [20]. However, it is specifically the proprioceptive neurons of the DRG that are deemed to be of particular interest in FRDA pathology and have recently become subject to further protocol optimisation. The DRG is composed of many PSN cell types, including nociceptors, thermoreceptors and mechanoreceptors. Comprising only 7.5% of PSNs found in the DRG, proprioceptive neurons are known to be particularly vulnerable in FRDA and have been shown to express the majority of the *FXN* found in the DRG [27, 28]. Thus far, almost all protocols that have been developed for the generation of PSNs from iPSCs, have focused on deriving nociceptor-enhanced cultures or mixed neuronal populations with few proprioceptors.

More recently, a novel protocol has described the generation and enrichment of PSN cultures for proprioceptors, starting with FRDA iPSCs. By modifying a pre-existing protocol for the generation of nociceptors, cultures significantly enriched for proprioceptors, shown to represent 50% of end-differentiated neurons, were successfully obtained. Utilising fluorescent-activated cell sorting, it is possible to further purify cultures to derive almost pure populations of proprioceptors [13]. This study describes, at the time of writing, the most phenotypically relevant iPSC-derived 2D neuronal model for FRDA. As technology continues to progress, attention within the iPSC field is now shifting in an attempt to create iPSC-derived 3D organoid models of neurodegenerative diseases. This has just recently been demonstrated by the generation of a 3D DRG organoid model of FRDA, which displays a transcriptional profile comparable to the native DRG and is composed of the representative PSN cell types [12].

### Utilising iPSC-derived neurons to investigate FRDA pathology

In addition to optimising and developing neuronal cell models for the characterisation of FRDA phenotypes, iPSC-derived models have also been used to further investigate FRDA pathology. There is still much to understand as to how the trinucleotide expansion drives the disease phenotype and several studies have been published utilising various FRDA iPSC-derived neurons to better understand mechanisms underpinning the disease. One such mechanism is the potential role of MMR proteins in *FXN* gene repression. The first study to generate FRDA iPSCs also crucially provided evidence for the potential role of MMR enzyme MSH2 in repeat instability [8]. A follow-up study investigated the expression of several different MMR enzymes in FRDA fibroblasts, FRDA iPSCs and iPSC-derived neural precursors. The study found an increased expression of MSH2, MSH3 and MSH6 enzymes in FRDA iPSCs when compared to fibroblasts and iPSC-derived neural stem cells. Additionally, the authors demonstrated that shRNA silencing of *MSH2* and *MSH3* hindered the GAA repeat expansion, strongly implicating a pathological active MMR system and an embryonic nature underlying the trinucleotide expansion [19]. This finding was strengthened by reports of high levels of GAA instability in FRDA-derived iPSCs, which corresponded with the upregulation of MMR enzymes MSH2, MSH3 and MSH6 [10]. Interestingly, reports of comparable levels of MHS2 and MSH6 in FRDA iPSC-derived neurospheres compared to FRDA iPSCs in the same study, contrasted reports of lower expression levels of these MMR enzymes in neural crest cells compared to corresponding iPSCs by another group [19]. Thus, while these findings demonstrate the potential role of MMR proteins in *FXN* gene repression, they also hint at other factors likely driving GAA-repeat instability.

Many other studies have also utilised iPSC-derived neuronal models to probe FRDA pathology and highlight the possibility of potential future therapeutic avenues. These include illustrating the potential of incretin-analogue therapy by investigating the key mediators of frataxin-deficiency-induced apoptosis [22], identification of potential protein targets and mechanistic pathways of interest for epigenetic therapy [21], determination of a previously undescribed differentially methylated region upstream of the GAA repeat expansion [23] and identification of differentially expressed glycolysis pathway genes associated with FRDA, capable of rescue with lentiviral *FXN* expression [25].

### iPSC-derived cardiomyocytes

Cardiomyocytes present another mature cell type particularly susceptible to the complex interplay of frataxin deficiency, aberrant iron homeostasis and mitochondrial dysfunction, resulting in the heart being a significant site of clinical pathology in FRDA. The majority of FRDA patients develop cardiomyopathy during their lifetime, with subsequent cardiac failure representing their most common cause of death [29]. Thus, alongside neurons, cardiomyocytes are a second major cell type of focus for studies investigating FRDA pathology. The first report of FRDA iPSC-derived cardiomyocytes was published in the same 2011 paper which also first detailed the generation of FRDA iPSC-derived neurons [9]. These cardiomyocytes were derived using a 2-step process, the first of which was the formation of embryoid bodies (EB) from pluripotent cells. Eight-day-old EBs were then cultured in specific conditions to induce cardiac differentiation, with cardiomyocytes being discernible by beating areas. Although there have been further developments in cardiomyocyte differentiation protocols beyond the initial 2-step process since this publication, progress has been less extensive as compared to neuronal models. This is likely due to the lack of heterogeneity of cardiac cell types susceptible to FRDA pathology alongside already well-established cardiomyocyte differentiation protocols. However, similar to the development of a 3D organoid model of the DRG, there has been one recent report detailing the development of a 3D cardiomimetic model. Utilising FRDA iPSC-derived ventricular cardiomyocytes, coupled with advanced engineered tissue platforms, Wong et al. were able to develop a 3D FRDA ventricular model to investigate the contractile and electrophysiological properties of the disease. When comparing their pathological models to healthy control constructs, they found electrophysiological defects consistent with clinical disease, as well as observing a positive correlation between *FXN* expression and contractility [11].

### Utilising iPSC-derived cardiomyocytes to investigate FRDA pathology

FRDA iPSC-derived cardiomyocytes have been utilised in several studies in an attempt to better understand the biological mechanisms that drive FRDA pathology. One of the first studies investigating disease phenotypes in an FRDA iPSC-derived cardiomyocyte model found that when compared to control lines, FRDA cardiomyocytes displayed characteristics of FRDA pathology, such as an increased frequency of compromised mitochondrial homeostasis and a larger abundance of dark respiration-compromised mitochondria [10]. Further characterisation of electrophysiological dysfunction reported that FRDA-derived cardiomyocytes exhibit an increase in beat rate variability in comparison to control lines. These disease phenotypes were reversed when cells were treated with nifedipine (an* L*-type Ca^2+^ channel inhibitor), implicating a Ca^2+^ handling deficiency in the electrophysiological impairment underpinning cardiac FRDA pathology [17]. A more recent study has utilised a novel isogenic model to investigate FRDA cardiomyopathy, by knocking down frataxin in iPSC-derived isogenic cardiomyocytes post-differentiation. Using transcriptomic analysis, it was demonstrated that unsurprisingly mitochondrial pathways were amongst the most affected by frataxin deficiency, further evidenced by the observed mitochondrial electron transport chain dysfunction. Interestingly, it was also shown that a type 1 interferon activation response was associated with frataxin knockdown, potentially mediated by an increase in cytosolic mitochondrial DNA [24]. Another recent study utilising transcriptome profiling and differential gene expression analysis has also identified genes involved with extracellular matrix functionality as significantly affected by FRDA pathology in iPSC-derived cardiomyocytes [25].

Another area of interest has been utilising cardiomyocyte models to investigate the pathological role of aberrant iron homeostasis, which is thought to be a key driver of clinical cardiac pathology seen in the disease. FRDA iPSC-derived cardiomyocytes display an accelerated cardiomyopathy phenotype when subjected to iron overloading conditions, with reports of hypertrophic changes, decreased ATP production and aberrant calcium handling in FRDA cardiomyocytes cultured in the presence of excessive iron supplement [14]. More recently, further insights have been provided into cardiac iron homeostasis in FRDA, with findings implicating a dysfunction of the hepcidin-ferroportin axis in diseased cells [18].

### iPSC-derived pancreatic β cells

As frataxin is an essential protein constitutively expressed by all cells within the body, organ systems outside the CNS and the heart are also commonly susceptible to FRDA pathology. One such clinical manifestation is the high prevalence of diabetes in FRDA patients when compared to an aged-matched control population. Both impaired insulin secretion and insulin resistance have been reported in FRDA, with 40% of FRDA patients exhibiting impaired glucose tolerance [30] and around 20% of patients going on to develop type 2 diabetes [31, 32]. Pancreatic β cell dysfunction is a key driver of the development of diabetes in FRDA, with β cells being particularly susceptible to mitochondrial dysfunction and metabolic stress [33]. For this reason, β cells present another mature cell type of interest for researchers to investigate FRDA pathology. However, until recently the generation of β cells from an iPSC source had remained a largely challenging proposition [34]. Thus far, only one group has successfully generated and utilised FRDA iPSC-derived β cells within the FRDA field. An FRDA β cell model that expresses differentiation markers consistent with β cell development and comparable to EndoC-βH1 β cells and human islet cells, has been successfully derived by employing a 7-stage differentiation protocol. When treated with the glucagon-like-peptide-1 analogue exenatide, FRDA iPSC-β cells upregulate frataxin expression, and show improved metabolic function and increased mitochondrial activity, demonstrating that iPSC-β cells can be utilised to investigate FRDA cellular phenotypes and potentially develop novel therapeutics [35].

### iPSC-derived retinal pigment epithelial cells

There are clear clinical implications for the visual system in FRDA, with several studies describing the presentation of ophthalmic manifestations in FRDA patients [36–39]. Optic neuropathy represents the most common manifestation, yet interestingly FRDA differs in presentation from other classical mitochondrial optic neuropathies. While optic nerve degeneration [36–38, 40] and increased sensitivity of retinal ganglion cells to oxidative stress [39] have been implicated in symptom development, there is still little understanding of the pathophysiology driving visual defects in FRDA. One particular cell type of interest is the retinal pigment epithelium (RPE) layer, the pigmented cell layer responsible for photoreceptor nourishment and associated with iron accumulation and retinal degeneration. Due to the dysfunctional nature of iron homeostasis in FRDA, the role the RPE potentially plays in FRDA optic pathology has been studied. Utilising FRDA-derived iPSCs, one study has successfully generated RPE cells, which were then investigated for a number of phenotypic characteristics. Interestingly, these iPSC-RPEs displayed no significant differences in oxidative phosphorylation activity and exhibited normal phagocytosis in comparison to controls [16]. While these findings implicate that RPE pathology may not be of significance in the development of FRDA optic neuropathy, the ability to generate mature ophthalmic cell types from FRDA iPSCs provides hope for a better understanding of the pathological causes of visual defects in FRDA.

## Utilisation of iPSC-derived models for FRDA therapeutic development

### FRDA therapeutic development remains a challenge

Despite the shared and significant efforts of doctors, scientists, patients and advocacy groups, FRDA currently remains without a cure. Despite the recent FDA approval of Skyclarys (omveloxolone) for the treatment of FRDA [41], the disease remains in desperate need of novel therapies, with existing approaches focusing solely on symptomatic relief and maintaining functionality. The major difficulty of developing efficacious disease-modifying drugs for FRDA is likely due to two barriers. The first barrier is an incomplete understanding of the biological mechanisms that drive the trinucleotide expansion and underpin disease progression. As our understanding of FRDA improves, our ability may improve to identify novel and promising pharmacological targets. The second major obstacle to pharmaceutical development is the enormous discrepancy in translating preclinical targets from bench to bedside. This failure is, in part, due to challenges surrounding the phenotypic relevancy of traditionally used preclinical models, such as patient fibroblast lines, which display limited susceptibility to frataxin deficiency.

**Fig. 2:**
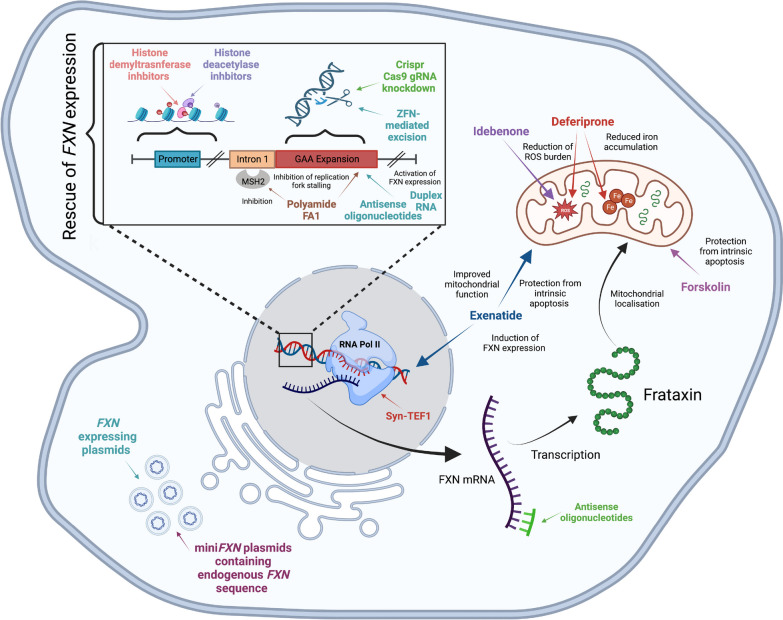
Visual representation of FRDA therapeutics investigated in FRDA iPSCs and their derived models. FRDA iPSCs have been utilised to generate phenotypically relevant cell models, providing a valuable platform for preclinical drug screening and development. Different therapeutic approaches have been studied using these novel models, with this figure detailing their approximate site of action within the cell. Modulation of the pathological FRDA gene has been investigated with epigenetic therapy, gene editing and other compounds such as polyamide FA1, antisense oligonucleotides and duplex RNA. Other approaches involve targeting the RNA Pol II enzyme and *FXN* mRNA transcript, as well as the delivery of exogenous plasmids carrying relevant *FXN*-expressing genetic information. As frataxin localises to the mitochondria, several therapeutics also act at the mitochondria to deal with the downstream consequences of frataxin deficiency, including reactive oxygen species (ROS) generation, iron accumulation and mitochondrial-activated apoptosis. Figure created with BioRender.com

The recent adoption of iPSC technology in the field of FRDA research has assisted in tackling both of these problems. Scientists can now derive a range of FRDA cell types of interest and use these preclinical models to study the biological mechanisms underpinning FRDA and to identify novel therapeutic targets. The advancement of differentiation protocols also allows access to the most phenotypically relevant cell types for preclinical testing, enabling iPSC-derived models to be used in the high-throughput screening of novel drugs. This section will explore how FRDA-iPSCs and their derived models have been used in several studies to accelerate the development of novel therapies for FRDA (Fig. 2), focusing on two major avenues: pharmacological approaches (Table 2) and gene therapy (Table 3).

**Table 2:** Studies utilising FRDA iPSCs and their derived models to investigate pharmacological approaches to FRDA therapy

Therapeutic strategy	Drug(s)	Study	Year	Drug class	Cell type(s) utilised	Study outcome
Pharmacological inhibition of epigenetic machinery	HDACi 109/RG2833	Soragni et al*.* [42]	2014	HDAC inhibitor	FRDA iPSC-derived neurons	HDAC inhibition by 5 µM of HDACi 109 upregulates *FXN* mRNA levels by approximately 2.5 folds in FRDA neurons
	HDACi 109 HDACi 966 HDACi 233	Soragni et al*.* [43]	2015	HDAC inhibitor	FRDA iPSC-derived neurons	Inhibition of both HDAC1 and HDAC3 is required for *FXN* activation. Only compounds targeting these HDACs are active in increasing *FXN* mRNA
	HDACi 109/RG2833	Codazzi et al. [44]	2016	HDAC inhibitor	FRDA iPSC-derived neurons	Significant increase in frataxin protein levels with no effect in neurons derived from healthy individuals
	Sodium butyrate (NaB) Tranylcypromine (Parnate)	Polak et al. [45]	2016	HDAC inhibitor Histone demethylase inhibitor (KDM1A inhibitor)	FRDA iPSCs	Both compounds correct some of the repressive histone modifications at the *FXN* locus exclusively in FRDA iPSCs and significantly increase *FXN* expression
	HDACi 109/RG2833	Hu et al*.* [46]	2017	HDAC inhibitor	FRDA iPSC-derived primary sensory neurons	FRDA PSNs treated with 5 µM of HDACi 109 resulted in a significant increase in both *FXN* mRNA and protein levels with no effect in control cells
	HDACi 109/RG2833	Lai et al*.* [47]	2019	HDAC inhibitor	FRDA iPSC-derived neurons FRDA iPSC-derived isogenic sensory neurons	HDACi treatment partially restores some cellular pathways affected by the loss of *FXN*. Those include neuronal function, regulation of transcription, extracellular matrix organization, and apoptosis
	Resveratrol Nicotinamide	Georges et al. [48]	2019	HDAC inhibitor	FRDA iPSC-derived neurons	Resveratrol and nicotinamide do not increase *FXN* expression in iPSC-derived neurons as they do on fibroblasts and lymphoblastoid cells
	CI994 Entinostat Mocetinostat	Schreiber et al. [49]	2022	HDAC inhibitor	FRDA iPSC-derived neural progenitor cells FRDA iPSC-derived neurons	Treatment with CI994, Entinostat and Mocetinostat increased FXN mRNA and protein levels in FRDA iPSC-derived NPCs and terminally differentiated neurons
DNA sequence-specific polyamides therapy	Polyamide FA1	Du et al*.* [19]	2012	β-alanine-linked polyamides	FRDAA iPSCs	FA1 partially blocks repeat expansion by displacing the mismatch repair enzyme MSH2 from intron 1 of *FXN*
	GAA-specific polyamide FA1	Gerhardt et al*.* [50]	2016	β-alanine-linked polyamides	FRDA iPSC-derived neurons	FA1 polyamide releases the replication fork stalling and alleviates expansion of the GAA repeats
Iron-homeostasis modulation	Idebenone (IDE) Deferiprone (DFP)	Lee et al*.* [51]	2016	Antioxidant coenzyme Q10 analogues (IDE) Iron chelators (DFP)	FRDA iPSC-derived cardiomyocytes	DFP modulates iron homeostasis and effectively relieves stress stimulation related to cardiomyopathy. DFP was also shown to be more effective than IDE for treating FRDA-mediated cardiomyopathy
Sequence-specific synthetic transcription elongation factor (Syn-TEFs) therapy	Syn-TEF1 Syn-TEF2	Erwin et al*.* [52]	2017	Syn-TEFs	FRDA iPSCs FRDA iPSC-derived neurons & cardiomyocytes	Syn-TEF1 stimulates the production of mature FXN protein by actively assisting RNA Pol II with productive elongation
Incretin receptor treatment	Forskolin Exenatide [D-Ala2]-GIP	Igoillo-Esteve et al. [22, 35]	2020	GLP-1 analogues	FRDA iPSC-derived sensory neurons FRDA iPSC-derived β cells	All 3 compounds increased frataxin protein expression in FRDA iPSC-derived neurons. Both Forskolin and exenatide decreased oxidative stress and inhibited the mitochondrial pathway of apoptosis. Exenatide was also shown to improve mitochondrial function
*FXN*-mRNA stabilisation	Antisense oligonucleotides targeting *FXN* mRNA	Li et al*.* [53]	2021	Antisense oligonucleotides	FRDA iPSC-derived neural progenitor cells	Electroporation-mediated delivery of *FXN* mRNA-targeting oligonucleotides increased *FXN* mRNA expression in FRDA iPSC-derived neural progenitor cells

GLP-1, glucagon-like-peptide-1; NPC, neural progenitor cell

**Table 3:** Studies utilising FRDA iPSCs and their derived models to develop gene therapy approaches for FRDA

Technique	Study	Year	Cell type(s) utilised	Study outcome
Zinc finger nuclease (ZFN)-mediated excision	Li et al*.* [80]	2015	FRDA iPSC-derived neurons	FRDA fibroblasts are corrected by ZFN-mediated excision of the GAA expansion. This correction persists during iPSC reprogramming, with disease phenotypes being reversed in ZFN-corrected FRDA iPSC-derived neuronal cells
	Li et al*.* [69]	2019	FRDA iPSC-derived cardiomyocytes	FRDA iPSC-derived cardiomyocytes exhibit pathological lipid accumulation and cardiac hypertrophy expression signatures which are reversed upon correction of the *FXN* gene via ZFN-mediated excision
Electroporation of gene silencing nucleic acids (antisense oligonucleotide activators and duplex RNA)	Shen et al*.* [67]	2019	FRDA iPSC-derived neuronal progenitor cells	Electroporation of duplex RNA and antisense oligonucleotide activators into FRDA iPSC-derived neural progenitor cells activates *FXN* expression
CRISPR Cas9 gRNA-mediated knockdown	Mazzara et al*.* [12]	2020	FRDA iPSC-derived dorsal root ganglia organoid sensory neurons	Reversal of FRDA molecular and cellular phenotypes upon excision of the *FXN* intron 1 in a 3D-DRG iPSC-derived organoid model of FRDA
LbL particle-mediated *FXN* expression plasmid delivery system	Czuba-Wojnilowicz et al*.* [68]	2020	FRDA iPSC-derived sensory neurons	Treatment of an FRDA iPSC-derived neuronal model with multi-layered nano-particles delivering *FXN*-expressing plasmids, results in a 27,000-fold increase in *FXN* expression
Transfection of mini*FXN* plasmids containing endogenous *FXN* sequence	Li et al*.* [81]	2020	FRDA iPSC-derived neurons FRDA iPSC-derived cardiomyocytes	Establishment of minimal endogenous promotor sequence required for *FXN* expression. Constructs containing this *FXN* expression control region resulted in successful *FXN* expression in FRDA iPSCs, which persisted during subsequent differentiation to FRDA iPSC-derived cardiomyocytes and neurons

### iPSC technology and FRDA pharmacological approaches

Several different pharmacological approaches have been investigated for the potential treatment of FRDA. The majority of preclinical in vitro studies which have utilised FRDA iPSCs and their derived models to test therapeutic agents have focused on epigenetic-based therapy (Table 2); however, several other exciting avenues have been explored.

#### Epigenetic pharmacotherapies for FRDA

The reprogramming of somatic cells into pluripotent stem cells has also opened the possibility of testing potential therapies on cells difficult to access, such as neurons or cardiomyocytes. In 2003, it was reported that expanded GAA·TTC repeats induce the formation of repressive heterochromatin in vivo and that the heterochromatin-mediated silencing might have a role in the regulation of *FXN* [54].

The inhibition of histone deacetylases (HDACs) emerged in 2006 as a possibility of alleviating *FXN* epigenetic repression to restore its expression. This therapeutic strategy has been widely studied for the past 15 years. It has been shown that HDAC inhibition by 5 µM of the HDAC inhibitor (HDACi) 2-aminobenzamide 109 upregulates *FXN* mRNA levels by approximately 2.5 fold in FRDA neurons [42]. Interestingly, the reversal of the *FXN* gene silencing in neurons derived from patient iPSCs is comparable to that found in previous studies performed on PBMCs and in vivo models [55–58]. Furthermore, the drug exposure that triggered epigenetic changes in their neuronal model in vitro is comparable to the exposure required in patients to induce epigenetic changes in circulating lymphoid cells and increases in gene expression. More in-depth studies from the same group investigating which HDACs are responsible for such an effect showed that inhibition of both HDAC1 and HDAC3 is required for *FXN* activation, and only compounds targeting these particular class I HDACs are active in increasing *FXN* mRNA [43].

Subsequently, an increase in frataxin protein level was also achieved following treatment of FRDA cortical neurons with 5 µM of HDACi 109, with no effect in neurons derived from healthy individuals [44]. Moreover, this study showed that *FXN* upregulation was accompanied by normalization of two Fe-S proteins, aconitase and NADH dehydrogenase [ubiquinone] Fe–S protein 3, in addition to lipoic acid-containing pyruvate dehydrogenase and 2-oxoglutarate dehydrogenase. All these proteins are severely reduced in FRDA neurons. HDACi 109 was also shown to normalise the levels of labile iron pool and ROS and almost fully protected FRDA neurons from oxidative stress-mediated cell death. The same group later derived PSNs as the most vulnerable and most affected cells in FRDA. Treatment of FRDA PSNs with 5 µM of HDACi 109 resulted in significant increases of *FXN* mRNA and protein with no effect in control cells [46]. Furthermore, an RNA-seq-based transcriptomic analysis of iPSC-derived CNS and isogenic sensory neurons found that some cellular pathways affected by the loss of *FXN*, such as neuronal function, regulation of transcription, extracellular matrix organization and apoptosis, were partially restored by HDACi 109 treatment [47].

Several other HDACis have been tested in iPSC-derived FRDA neurons, including resveratrol (a polyphenol which also acts as a sirtuin activator) and nicotinamide [48]. FRDA iPSCs have also been used to test various small molecule inhibitors that target other epigenetic machinery, including DNA methylation, histone acetylation, and histone methylation [45]. Reprogramming FRDA fibroblasts in the presence of sodium butyrate, an HDAC class I inhibitor, or tranylcypromine, a lysine-specific demethylase 1 inhibitor, significantly increased *FXN* expression and was associated with the correction of some of the repressive histone modifications at the *FXN* locus in derived FRDA iPSCs. When these FRDA iPSCs were subsequently differentiated into neurons, a re-silencing of *FXN* expression was observed.

Interestingly, a recent study has described the generation of a novel FRDA reporter cell line created by *FXN*-Nanoluciferase (*FXN*-NLuc) infusion into FRDA iPSCs followed by differentiation into neural progenitor cells (NPCs). Utilising *FXN*-NLuc NPCs to screen a library of 281 compounds, three HDACis (CI994, Entinostat and Mocetinostat) were shown to increase the relative levels of *FXN* mRNA and protein. These results were further reproducible when treating iPSC-derived terminally differentiated neurons [49]. This study provides proof of concept that the generation of novel FRDA iPSC-derived reporter cell lines is capable of accelerating drug discovery and development due to their phenotypic relevance.

#### Investigation of non-epigenetic pharmacological approaches for FRDA

GAA-specific polyamides have been also tested as potential therapeutic approaches using iPSCs derived from FRDA patients. Several studies have reported that expanded GAA·TTC repeats adopt unusual non-B DNA structures, such as triplexes and intramolecular sticky DNA [59–65]. Ohshima and colleagues documented that triplexes and/or sticky DNA block elongation by RNA polymerase II (RNA Pol II) [59]. Therefore, molecules that reverse triplex or sticky DNA in the frataxin gene may be considered therapeutic. The specific pyrrole-imidazole polyamide FA1 binds GAA·TTC tracts with high affinity, disrupts the intramolecular DNA·DNA-associated region of the sticky-DNA conformation and relieves transcriptional repression of the *FXN* gene in FRDA cells [66]. It has also been shown that FA1 at 5 µM impedes GAA·TTC triplet-repeat expansion in FRDA iPSCs and that the stabilization of the expanded GAA repeats could be due to the release of the replication fork stalling [19, 50], suggesting that DNA triplexes and fork stalling may be a potential therapeutic target for FRDA.

Driving the triplex structure conformations towards the B-form DNA conformation may not be enough to elicit sufficient *FXN* expression. Actively assisting productive RNA Pol II elongation has also emerged as a novel strategy to increase *FXN* expression [52]. It has been shown that synthetic transcription elongation factors can stimulate *FXN* expression in iPSCs, cardiomyocytes and neurons derived from FRDA patients, in addition to other cellular and in vivo models [52]. These synthetic molecules are programmable DNA binders that target desired genomic loci and at the same time engage the transcription elongation machinery. Interestingly, these molecules use the pyrrole-imidazole polyamide as a DNA binder, the same polyamide used by Burnett and collegues in 2006 to engage the GAA·TTC tracts, suggesting the potential of this structure as a high-affinity binder. Another interesting approach involves *FXN* mRNA transcript stabilisation to increase protein expression. This has recently been demonstrated using *FXN* mRNA-targeting antisense oligonucleotides (ASOs) capable of increasing *FXN* mRNA expression in FRDA iPSC-derived NPCs [53].

Rather than increasing frataxin expression, models derived from FRDA iPSCs have additionally been utilised to assess the downstream effects of frataxin deficiency, such as increased oxidative stress or aberrant iron metabolism. FRDA iPSC-derived cardiomyocytes were used to assess the efficacy of the antioxidant idebenone and the iron chelator deferiprone. It was reported that deferiprone was able to suppress the synthesis of ROS and improve the cardiac electrical-contraction coupling function [51]. The authors of this study concluded that deferiprone modulates iron homeostasis in FRDA cardiomyocytes and effectively relieves stress stimulation related to cardiomyopathy. Furthermore, the cAMP inducer forskolin, and incretin analogues [D-Ala2]-GIP and exenatide, reduce apoptosis in frataxin-deficient β cells and neurons, through decreasing oxidative stress and inhibiting the mitochondrial pathway of apoptosis [22]. Moreover, exenatide has been shown to induce frataxin expression, improve mitochondrial function and reduce oxidative stress in FRDA patient iPSC-derived β cells and sensory neurons, implicating incretin receptors as potential therapeutic targets in FRDA [35].

### iPSC technology and gene therapy for FRDA

Another promising therapeutic avenue for the treatment of FRDA is gene therapy. Unlike some of the previously explored pharmacological methods, a gene therapy approach aims to cure patients directly, rather than acting as a chronic treatment. This is achieved by modulating the expression of key genes to restore function, either by direct excision or more commonly, by delivery of a wild-type transgene to patient cells using adeno-associated viruses (AAVs), retroviruses or herpes simplex virus type 1. Recently, several non-viral vector approaches of delivery have also been explored, including electroporation, zinc-finger nucleases (ZFNs) and layer-by-layer (LbL) particles [67–69]. Gene therapy has already been investigated as a promising avenue for many other neurodegenerative diseases including Parkinson’s, Alzheimer’s and Huntington’s, with encouraging results [70–73]. More recently, the field has also attempted to tackle rarer genetic disorders such as FRDA. As FRDA is caused by a dysfunction in a single gene, it presents a promising and conceptually simple candidate disorder for gene therapy. It is thought that delivery and expression of the *FXN* gene will complement the loss of *FXN* expression from the mutant alleles and potentially reverse FRDA phenotypes. Several preclinical gene therapy studies for FRDA have been performed, with current approaches focusing on the delivery of vectors driving *FXN* gene expression[27, 74–79]. Some of these studies have returned promising results, demonstrating an improvement of FRDA phenotypes in cell and animal models and reinforcing the prospect of gene therapy as a potential long-term cure for FRDA. The advent of iPSC technology has been instrumental in developing the field of gene therapy for FRDA, with studies utilising FRDA iPSC-derived models to investigate potential gene targets as well as exploring the effectiveness of various vectors of delivery (see Table 3).

#### Gene therapy-mediated excision of FRDA expansion

One of the earliest efforts to correct FRDA pathology using a gene therapy approach is the utilization of engineered nucleases to specifically target and excise the pathological expanded GAA repeat in the *FXN* intron 1 region. Li et al. reported correction of the FRDA genetic defect as well as restoration of *FXN* expression and FRDA biomarkers in patient fibroblast and lymphoblast cell lines, by employing simultaneous cleavage with two zinc finger nucleases (ZFNs). These GAA-corrected patient fibroblasts were then subsequently reprogrammed to FRDA iPSCs and further differentiated into neurons, to investigate the effects of genetic reprogramming in a more phenotypically relevant cell type. The authors found that the phenotypic correction persisted in the iPSCs and their derived neurons, with GAA-corrected neurons expressing roughly threefold frataxin in comparison to control non-GAA-edited neurons derived from non-GAA-edited iPSCs [80]. These results highlight the therapeutic potential of a ZFN approach, indicating that editing a single allele in patient cells is potentially enough to correct the FRDA molecular phenotype. Additionally, the study described a new method to investigate FRDA pathology, utilising genetically corrected isogenic cell lines with the potential to better resemble control-patient counterparts in future studies. A follow-up study utilised FRDA iPSC-derived cardiomyocytes which demonstrated many features characteristic of cardiomyopathy, including a decrease in *FXN* expression and the pathological accumulation of lipids. The ZFN gene-editing approach was also able to reverse these pathological phenotypes in isogenic cardiomyocyte lines [69]. The results of these two studies and the ability to derive isogenic cell lines provide exciting evidence for the use of gene-editing technology in the treatment of FRDA, as well as the potential for transplantation therapies, through the replacement of diseased cells with specific patient-corrected cell lines.

The potential of the frataxin gene-editing approach has also been explored utilising a 3D organoid model of an FRDA DRG. Exploiting CRISPR/Cas9-mediated excision, the successful rescue of phenotypes in DRGs derived from gene-edited FRDA patient iPSCs has been reported. Interestingly, deletion of just the expanded GAA repeats resulted in only a mild increase in *FXN* mRNA expression and protein levels, while the additional excision of almost the entire surrounding *FXN* intron 1 region showed a more significant rescue of frataxin levels [12]. These results are in keeping with numerous studies that have highlighted the high number of repressive histone marks within the chromatin of *FXN* intron 1, implicating that removal of the entire *FXN* intron 1 region may be required to act as the optimal therapeutic target for future gene-therapy approaches [82, 83].

#### Gene therapy-mediated modulation of *FXN* expression

In addition to excising the pathological trinucleotide expansion, an alternative gene therapy approach involves modulation of the *FXN* gene by targeted delivery of nucleic acids and other compounds capable of activating functional frataxin expression. While this is a promising approach, more work is required to understand the regulatory nature of the non-coding sections of the *FXN* gene, to elucidate the optimal region to target with gene therapy. To better understand how we might stimulate the *FXN* gene, a recent study evaluated which regions of the *FXN* promoter sequence were essential for gene expression. After creating constructs of varying *FXN* sequence lengths, they utilised site-specific integration in FRDA iPSCs in the hope to define an endogenous *FXN* promotor sequence indispensable for frataxin expression in humans. The group was successfully able to report on such a sequence, demonstrating *FXN* expression in the treated FRDA iPSCs and their neuronal and cardiomyocyte derivatives. When utilising the same constructs to deliver AAV-expressing mini *FXN* genes to mice, they were further able to show activation of frataxin expression in vivo [81]. These findings help decipher and define optimal targets within the promotor region of *FXN*, and provide evidence for the viral delivery of endogenous *FXN* sequences to restore frataxin expression as a potential gene therapy approach for FRDA.

Limitations of viral delivery within the CNS, including immune implications, low transfection rates, restricted genetic carrying capacity and undesirable off-target effects, have resulted in the establishment of several unconventional, yet innovative non-viral methods of delivery for gene therapy. Recently, two studies have been published utilising FRDA iPSCs and their derivatives to help develop and test novel delivery techniques for FRDA gene therapy [67, 68]. The first of these established a rapid and reproducible protocol for the delivery of nucleic acids into FRDA iPSC-derived NPCs using electroporation, a technique which increases the permeability of the cell membrane to drugs and other compounds. Utilising this novel method for the delivery of ASOs, a threefold increase in *FXN* protein expression in FRDA iPSC-NPCs was obtained, with expression levels similar to corresponding wild-type controls. These findings describe the use of the MaxCyte electroporation system, which notably presents a comparatively simpler and less toxic alternative to other traditionally used transfection methods. Additionally, the ability to successfully apply this protocol to FRDA iPSC-NPCs and demonstrate the ability of ASOs to activate *FXN* expression, provides exciting evidence for the potential of synthetic oligonucleotides in FRDA gene therapy [67]. Interestingly, a recent study has also shown that ASO-mediated end targeting may also provide a promising approach for *FXN* upregulation, via the extension of *FXN* mRNA half-life [53].

Another exciting and novel delivery approach involves the use of multi-layered nano-particles for the delivery of *FXN-*expressing plasmids. These particles are assembled using an LbL approach and demonstrate effective cytoplasmic uptake in FRDA iPSC-derived sensory neurons following a 24-h incubation period. Additionally, sensory neurons treated with LbL particles bound with *FXN*-expressing plasmid displayed a 27,000 fold increase in *FXN* expression when compared to non-plasmid-bound treated controls [68]. These findings emphasize the theoretical potency of alternative non-viral methods of gene therapy, while also highlighting the potential of an *FXN*-expressing plasmid approach for the treatment of FRDA.

## Future directions

Since the introduction of iPSC technology in 2006, there have been over 35 studies which utilised FRDA iPSCs and their derived models, at the time of writing, within the field of FRDA research. These novel models have been applied to investigate the molecular mechanisms underpinning FRDA pathology, as well as for the advancement of new therapeutic avenues within FRDA drug development and gene therapy. It is hoped that the combination of a deeper understanding of disease pathogenesis with improved cellular models may eventually lead to the development of disease-modifying strategies for the disorder. Despite the exciting prospect of these novel models, they are not without limitations. This section will touch on some of the key considerations for current FRDA iPSC research, as well as exploring some of the potential future directions the field may take.

### A representative phenotype?

One particular area of contention is the ability of FRDA iPSCs and their derived models to exhibit overt and measurable phenotypes representative of FRDA pathology. Several studies have reported various empirical phenotypic traits in FRDA iPSC-derived neurons and cardiomyocytes. These measurable phenotypes provide a predictive model which can be utilised for in vitro drug discovery. Interestingly, however, several reported FRDA phenotypes are seen to be relatively mild when compared to control cell lines. Additionally, other studies have reported a failure to observe characteristic FRDA phenotypes in their iPSC-derived models. For example, in an aforementioned study, neural derivatives generated from FRDA iPSCs were found to give rise to functional neurons, with no apparent signs of FRDA pathology. When FRDA iPSC-generated neurons were compared with their controls, no significant differences in the levels of cell death or mitochondrial volume and function were seen. Further examination of the in vivo differentiation potential of these FRDA iPSC-derived neural progenitors, revealed an ability to integrate and survive within the cerebellum of adult mice, giving rise to both neurons and glial cells [15]. Additionally, FRDA iPSC-derived cardiomyocytes have been shown to be comparable in size to wild-type controls, demonstrating no signs of dysfunctional ATP production or calcium handling. It was only after these cells were cultured with an additional exogenous Fe^2+^ supplement of 200 µM, that any discernible phenotypic differences emerged [51]. Notably, such high levels of endogenous iron-overloading are highly unlikely to be seen in healthy cardiomyocytes, bringing the physiological relevance of these findings into question.

One potential reason for the discrepancy in the phenotypic presentation of FRDA iPSC-derived cells may be due to the intrinsic differences in their repeat expansion lengths. The severity of disease phenotype is known to be correlated with *FXN* production, which has been associated with the number of pathological GAA repeats [84, 85]. Over 95% of FRDA patients have bi-allelic expansions ranging from 66 to 1700 repeats, with the majority of symptomatic patients possessing 600–900 repeats [86, 87]. It is thought that FXN protein expression of approximately ~ 5%–35% of normal levels is required for FRDA patients to display symptoms [88–91]. Published studies which reported differences in their observed phenotypes, utilised FRDA patient cells differing in the size of their repeat expansions to generate FRDA iPSCs and their derived models. Thus, it is likely that differences in repeat length between patient cell lines influenced levels of FXN protein expression in these models and consequently their phenotypic presentation of FRDA pathology. The problem of line-to-line variability may be addressed with the use of isogenic cell lines, allowing the creation of genetically modified homogenous cell lines which better resemble healthy counterparts.

Additionally, it is important to note that FRDA is a late-onset childhood disorder, with symptoms tending to present between the ages of 5–15. Often neurons and cardiomyocytes derived from FRDA iPSCs and used for experimentation are immature, with a neonatal resemblance, therefore limiting the ability of these models to accurately recapitulate disease phenotypes. This is due to the reprogramming process, which resets the developmental clock of donor patient cells back to a stem cell state. These subsequent iPSC-derived models may not demonstrate overt FRDA phenotypes until they have matured to a later stage in their growth, or have had the required time to be adequately exposed to potential environmental stressors or accumulate the progressive degenerative pathology. Potential solutions for this issue include generating artificially-aged neurons through the direct lineage conversion of patient fibroblasts to create induced neurons (iNs) [92, 93], or actively stimulating the rapid ageing of iPSC-derived models via progerin exposure [94]. A recently published study has also described a novel differentiation method using Sfrp2 to induce mature cardiomyocytes from human iPSCs [95].

Neuronal degeneration and phenotypic presentation may also be specific to particular neuronal subpopulations. The majority of studies investigating phenotypes in FRDA iPSC-derived neurons have generated and utilised central sensory neurons, mixed, or non-specific nociceptor predominant cultures of PSNs. As these neuronal subtypes are less vulnerable to frataxin deficiency, their exhibited pathological phenotypes may consequentially be mild and/or difficult to detect. Although it has been shown that proprioceptor-enhanced cultures of FRDA iPSC-derived neurons can be generated [13], further work is required to establish whether this vulnerable neuronal subpopulation displays more pronounced and representative phenotypes of FRDA pathology. Reports on the ability to observe and reverse phenotypes such as defective survival and morphology, impaired Fe–S cluster biogenesis and defective axonal mitochondrial behaviour in a 3D iPSC-derived DRG model of FRDA [12], provide further hope for the use of novel iPSC-derived FRDA neuronal models and their phenotypes for the study and development of potential therapies.

### Deriving neurons of the cerebellum: the final elusive cell type?

Alongside peripheral nerves and the dorsal root ganglion, the cerebellum presents another major site within the CNS severely affected by the neuropathology of FRDA [96]. The dysfunction and degeneration of neurons of the deep cerebellar nuclei give rise to gait ataxia, progressive loss of motor coordination, dysarthria and saccadic eye movement alterations frequently seen in FRDA patients [97]. Of particular interest are Purkinje cells, which constitute the sole neural output in the cerebellar cortex. Despite numerous studies detailing established protocols for the derivation of cortical and PSNs from FRDA-iPSCs, there have been no reports of the generation of FRDA iPSC-derived neurons of the deep cerebellar nuclei, or Purkinje cells which modulate their activity. Even outside of the field of FRDA research, only a handful of studies have successfully utilised pluripotent cells for the generation of Purkinje cells [98–100], with just a single study reporting successful derivation from patient iPSCs [98].

The generation of Purkinje cells has remained largely elusive likely due to their lengthy and challenging differentiation protocols, as well as their complex morphological characteristics making them difficult to differentiate and culture [101, 102]. Successfully deriving Purkinje cells from FRDA iPSCs remains a key hurdle the field must overcome. Recent advancements such as the reports of a simplified and more reproducible Purkinje cell differentiation protocol [103] and the ability to mature pluripotent stem cell-derived cerebellar neurons without co-culture [104], mean that the generation of FRDA iPSC-derived Purkinje neurons is one step closer to reality. Such models have the potential to provide insight into cerebellar dysfunction in FRDA and  may be of huge utility for the development of novel therapies. If and when it is possible to generate FRDA iPSC-derived Purkinje neurons of the cerebellum, it may also be of particular interest to look at differences between affected and unaffected neurons, to determine which characteristics prompt certain neurons to be susceptible to frataxin deficiency.

### Generation of FRDA isogenic cell lines

Early studies on iPSCs have relied on using cells that are aged-matched, unaffected by the pathology and with the same family pedigree as controls. However, despite the best efforts to match patient and control cells, there are differences in genetic background, single nucleotide polymorphisms, and other confounders that make comparisons between them sometimes hard to interpret. The rapid development of genome editing techniques has made possible the creation of isogenic lines. These lines are generated from the same individual and have been engineered to differ only at one specific locus, in this case, the GAA expansion at intron 1 of the *FXN* gene. External to expansion length, cells are genetically identical, overcoming in this way, inter-individual variabilities in all types of studies including genome-wide studies. For the majority of FRDA isogenic cell lines, expansion length is genetically modified prior to iPSC induction and any potential further differentiation. Interestingly, a recent study has also reported the ability to generate a novel isogenic model of FRDA by directly knocking down frataxin expression in FRDA iPSC-derived cardiomyocytes [24]. By modifying genes after differentiation, this technique further controls for variables caused by the independent processing of isogenic pairs, including passage number and any potential variance which occurs during the differentiation process, such as the cellular composition of obtained cultures. The use of isogenic cell lines has also been fundamental for disease modelling, for the understanding of disease biology and more recently, for testing novel therapeutic agents. The ability to specifically alter the length of the GAA expansion may also provide researchers with a potential tool to theoretically investigate how repeat length influences treatment response to therapy, in otherwise genetically identical cell lines. With time, more studies will only use isogenic cell lines as controls, and these important tools will become the must-have controls in a wide variety of studies.

### Limitations of 2D cultures and the development of organoid modelling

For decades, cell culture has been central in advancing our understanding of cell biology principles, tissue and cell morphology, mechanisms of disease, and the action of drugs. It is a widely used technique that up until recently, has mainly relied on 2D cultures. There are many advantages of using this type of culture including that they are well established, they are mostly inexpensive to maintain, the data generated with them are vast and allow the comparison between studies and they are easier to understand. However, they do not recapitulate the in vivo environment where they grow, the interaction between different cell types is usually perturbed, and they do not offer a physiologically relevant spatial context. These and other limitations have been key to the fast-growing field of 3D cultures. The capability to model cells in vivo while being cultured in vitro has become one of the key drivers of the adoption of 3D cultures in many laboratories. When speaking about the brain, it is clear why 3D cultures are being increasingly developed and used. The brain being an extremely complex 3D arrangement of cells cannot be modelled properly in 2D. Moreover, certain research questions cannot be addressed using a 2D cell culture system. However, it is important to keep in mind that 3D cultures are much more heterogeneous and complex and they are challenging to standardize and analyse.

3D neuronal cultures are not new in FRDA. Neurospheres, one of the earliest 3D neuronal cultures, have been used by many groups over the years. However, more recently the tendency has been to move to more complex systems such as organoids. Recently, DRG organoids were generated by in vitro differentiation of FRDA iPSCs [12]. These 3D DRG-like structures were generated by culturing single iPSC aggregates in low-adhesion 96-well plates for 16 days in vitro and exposing them to a sequential combination of small molecules that differentiate iPSCs into neural crest neuronal derivatives [12]. Initially, the authors of the study tried to generate a 2D culture of sensory neurons from FRDA patient iPSCs and encountered extensive cell death. They hypothesized that the absence of glial cells and the lack of sufficient cell-to-cell contacts in a 2D system could explain the reduced viability. Their new 3D arrangement of cells proved to be, at the transcriptomic level, similar to primary DRGs, exhibiting a global transcriptional reconfiguration towards sensory neuronal cells, as well as supporting peripheral glial cells. Additionally, they better model the spatial organization and cellular composition when compared to classical 2D differentiated neuronal cultures.

Engineered human cardiac models of FRDA have also been reported [11]. iPSCs have been used in a pioneering study to generate both 3D human ventricular cardiac anisotropic sheets and ventricular cardiac tissue strips, useful for measuring electrical conduction as a syncytium and evaluating contractility, respectively. These cardiomimetic in vitro model systems possess the ability to produce multicellular readouts such as conduction velocity and magnitude of force generation, not possible to obtain in single-cell models. When it comes to a complex arrangement of cells such as those in different parts of the brain or the heart, it is increasingly useful to understand these systems as a whole, to more accurately translate research to a clinical setting.

### An ideal paradigm of FRDA drug discovery

Drug discovery and development have been performed for the past few decades using immortalized cell lines, as well as primary cells derived from patients and animal models. The discovery of iPSCs opened a new avenue for the study of otherwise inaccessible human cells in vitro, presenting a novel platform for drug testing. In FRDA, we can now study neurons and cardiomyocytes, two historically inaccessible cell types disproportionately affected by frataxin deficiency. Moreover, differentiation protocols are becoming more specialized, allowing the derivation of precise cellular subpopulations. This particular characteristic is important for addressing specific questions where a mixed culture may create confounders. iPSCs can also provide an extra layer of safety to assess toxicity and teratogencity before advancing drugs to lengthy and expensive clinical trials.

**Fig. 3:**
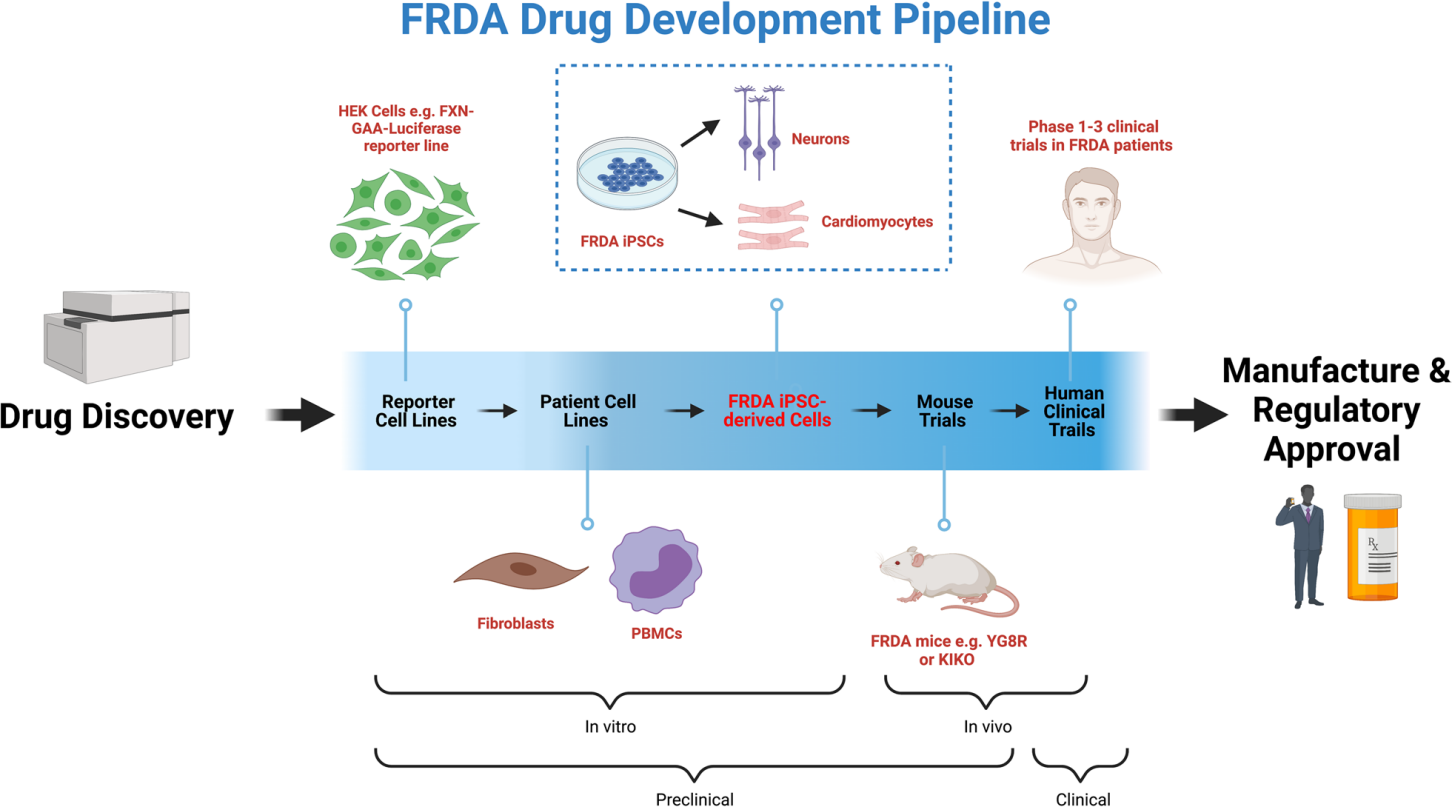
An ideal paradigm of drug discovery utilising patient iPSC-derived models. Following drug discovery and screening, potential FRDA therapeutic compounds undergo in vitro testing in reporter cell lines and patient cell lines before traditionally progressing to in vivo mouse and human clinical trials. We propose the additional testing of compounds in patient iPSC-derived models (e.g., cardiomyocytes and neurons), before in vivo and clinical studies. The addition of this step allows the assessment of drugs in a phenotypically relevant and human-specific model of FRDA, screening out drugs which may fail to show therapeutic benefit in expensive and time-consuming human clinical trials. An alternative to disease phenotype-driven drug screening may involve high-throughput screening of drugs directly in iPSC-derived cells with *FXN* levels as the read-out, such as by using iPSC-derived FRDA lines or iPSC *FXN-GAA* reporter models. Figure created with BioRender.com

In order to advance potential therapeutic agents to clinical trials, it is vital to understand if these drugs reproduce their desired effect on the cell types most affected by FRDA pathology. Translating in vitro results into a clinical setting is already challenging and presents one of the key features of the poor predictability of drug efficacy during clinical trials. The use of cell types with little relevance for FRDA in the preclinical phase only further increases this uncertainty. This was the case with resveratrol, which performed well in fibroblasts and lymphoblastoid cells, but underwhelmingly in a subsequent clinical trial [105, 106]. Once resveratrol was tested on neurons derived from FRDA iPSCs, only a modest (roughly 1.3 fold) increase in *FXN* mRNA expression was observed 48 h following treatment with 25 µM resveratrol, with the drug failing to significantly upregulate frataxin protein expression [48]. Nicotinamide, another HDACi which had returned promising results in FRDA primary lymphocytes [107], also failed to significantly upregulate frataxin protein expression in FRDA neurons when tested in the same study [48]. With this in mind, we propose an ideal paradigm for drug discovery and development using iPSC-derived specific cell types in the preclinical validation of potential drugs (Fig. 3). This model emphasizes the importance of using iPSC-derived cells for preclinical modelling to select the most promising agents to progress to clinical trials, saving resources and increasing the potential of translational success.

### FRDA iPSCs for cell replacement therapy

Cell replacement therapy represents a novel and exciting therapeutic avenue currently being explored for FRDA. Transplantation studies have shown that human donor cells survive and functionally integrate into the CNS, and this successful integration can lead to damaged neural tissue repair [108]. However, most of the studies performed address diseases that affect the CNS, and fewer have been reported for the PNS. FRDA iPSC-derived neural progenitors transplanted into adult rat cerebellum not only survive but also integrate, and differentiate within the host tissue [15]. Furthermore, it has recently been demonstrated that FRDA iPSC-derived sensory neural progenitors can mature and integrate within the adult DRG in vivo. These grafted cells express neuronal sensory markers including nociceptors, mechanoreceptors, and proprioceptors [109]. Transplantation of haemopoetic stem and progenitor cells has been shown to rescue deficits in the YG8R mouse model of FRDA [110]. Furthermore, gene-corrected human hematopoetic stem cells have already been reported and provide a basis for autologous transplantation [111]. These studies demonstrate proof-of-principle that cell replacement may be a promising treatment strategy to replenish some of the most severely affected cell types in FRDA. By combining gene-editing technologies and transplantation therapy in the future it may be possible to offer FRDA patients the potential of transplanting sensory neuron or cardiomyocyte grafts where the GAA expansion has been removed. This may be achieved by creating isogenic corrected cell line grafts from FRDA patient iPSCs. While this type of scenario currently remains futuristic, promising and rapid advances in science may give life to this approach sooner rather than later.

## Conclusions

The recent advent of iPSC technology has revolutionised the study of FRDA. Utilising FRDA iPSCs, researchers are capable of generating phenotypically relevant and previously inaccessible cell models uniquely affected by FRDA pathology. These models allow researchers to bypass several drawbacks presented by historical cellular and rodent models of FRDA, providing them with a novel approach to investigating FRDA pathology and therapeutic development. In this review, we outline how iPSC technology has been utilised to advance the field of FRDA research over the past decades. We detail the generation of FRDA iPSCs from somatic patient cells, as well as their further differentiation into appropriate cell types vulnerable to *FXN* deficiency. We describe how these cell models have developed alongside the progression of novel differentiation protocols, and have been utilised for the characterisation of FRDA phenotypes and to investigate and model the pathological mechanisms underpinning disease development. We further go on to explore how FRDA iPSCs and their derived models provide an exciting platform for therapeutic development, being used to screen novel pharmacological and gene therapy approaches. We conclude by discussing some of the key considerations of iPSC technology and its future perspectives in the field of FRDA research. It is hoped that further utilisation and expansion of FRDA iPSC technology will accelerate the development of disease-modifying strategies for FRDA by enhancing our ability to study the disorder and improve the successful translation of preclinical drugs to in vivo studies.

## Data Availability

Not applicable.

## Abbreviations

FRDA: Friedreich ataxia
iPSC: Induced pluripotent stem cell
CNS: Central nervous sytem
PNS: Peripheral nervous system
ESC: Embryonic stem cells
MMR: Mismatch repair
PSN: Peripheral sensory neuron
DRG: Dorsal root ganglion
EB: Embryoid bodies
RPE: Retinal pigment epithelium
HDAC: Histone deacetylases
HDACi: Histone deactylase inhibitor
ROS: Reactive oxygen species
FXN-NLuc: FXN-Nanoluciferase
NPC: Neural progenitor cell
RNA Pol II: RNA polymerase II
ASO: Antisense oligonucleotides
AAV: Adeno-assocaited viruses
ZFN: Zinc-finger nuclease
LbL: Layer-by-layer
iN: Induced neuron
